# Proximal ADP-ribose Hydrolysis in Trypanosomatids is Catalyzed by a Macrodomain

**DOI:** 10.1038/srep24213

**Published:** 2016-04-11

**Authors:** Teemu Haikarainen, Lari Lehtiö

**Affiliations:** 1 Biocenter Oulu and Faculty of Biochemistry and Molecular Medicine, University of Oulu, FI-90014 Oulu, Finland

## Abstract

ADP-ribosylation is a ubiquitous protein modification utilized by both prokaryotes and eukaryotes for several cellular functions, such as DNA repair, proliferation, and cell signaling. Higher eukaryotes, such as humans, utilize various enzymes to reverse the modification and to regulate ADP-ribose dependent signaling. In contrast, some lower eukaryotes, including trypanosomatids, lack many of these enzymes and therefore have a much more simplified ADP-ribose metabolism. Here we identified and characterized ADP-ribose hydrolases from *Trypanosoma brucei* and *Trypanosoma cruzi*, which are homologous to human *O*-acetyl-ADP-ribose deacetylases MacroD1 and MacroD2. The enzymes are capable for hydrolysis of protein linked ADP-ribose and a product of sirtuin-mediated lysine deacetylation, *O*-acetyl-ADP-ribose. Crystal structures of the trypanosomatid macrodomains revealed a conserved catalytic site with distinct differences to human MacroD1 and MacroD2.

ADP-ribosylation is a covalent modification where one or multiple ADP-ribose units are attached to a target protein. In eukaryotes, the modification is catalyzed by poly-ADP-ribose polymerases (PARPs), silent information regulators (sirtuins), and poorly characterized membrane anchored arginine ADP-ribosyltransferases (ARTs)^1^,^2^,^3^. ADP-ribosylation regulates several cellular events, including DNA repair, cell cycle progression, transcription and cell death. In humans over 20 enzymes have been found to catalyze ADP-ribosylation and modification can be reversed by various enzymes: PARG (poly(ADP-ribose) glycohydrolase) and ARH3 (ADP-ribosylhydrolase 3), which can hydrolyze ADP-ribose polymer leaving the proximal ADP-ribose molecule attached to the modified protein; MacroD1 and MacroD2, which cleave the proximal mono-ADP-ribosyl; and OARD1 (*O*-acetyl-ADP-ribose deacetylase 1), which is capable of hydroxylation of the proximal mono-ADP-ribosylation and also cleaving the entire PAR *en bloc*^4^. There are also less characterized enzymes involved in ADP-ribose hydrolysis, namely ARH1, which hydrolyzes mono-ADP-ribosylated arginines^5^ and Nudix hydrolase 16, which is able to remove both mono-and poly-ADP-ribosylation leaving the modified protein with a ribose-5′-phosphate^6^. MacroD1, MacroD2, OARD1, and ARH3 have also *O*-acetyl-ADP-ribose hydrolysis activity as they are capable of removing the acetyl group from *O*-acetyl-ADP-ribose produced by sirtuin-mediated lysine deacetylation^7^,^8^,^9^.

*Trypanosoma brucei* and *Trypanosoma cruzi* are parasitic protozoa responsible for severe human and animal diseases. *T. brucei* is the causative agent of African trypanosomiasis or sleeping sickness and *T. cruzi* is responsible for South American trypanosomiasis or Chagas Disease. These parasites seem to have a highly simplified ADP-ribose metabolism compared to higher eukaryotes, such as humans. They contain a single PARP opposed to 17 PARPs found in humans^10^,^11^,^12^ and have two (*T. cruzi*) or three (*T. brucei*) sirtuins, while humans have seven (although not all have ADP-ribosylating activity)^13^. Only one ADP-ribose hydrolase, PARG, has been characterized from the parasites^14^. Recently, a phylogenetic analysis of proteins linked to ADP-ribose metabolism identified a single MacroD1/MacroD2 homologue in *T. brucei*^15^. We analyzed the available genome sequences and observed that besides PARG and MacroD1/MacroD2 homologue, *T. brucei* and *T. cruzi* do not have other known enzymes capable for the hydrolysis of ADP-ribose. We show that the trypanosomatid MacroD1/MacroD2 homologues are hydrolyzing both protein-linked ADP-ribose and free *O*-acetyl-ADP-ribose. The crystal structures of the enzymes reveal highly conserved macrodomain folds containing conserved ADP-ribose binding sites.

## Results

### Identification and characterization of trypanosomal proximal ADP-ribose hydrolases

**Figure 1:**
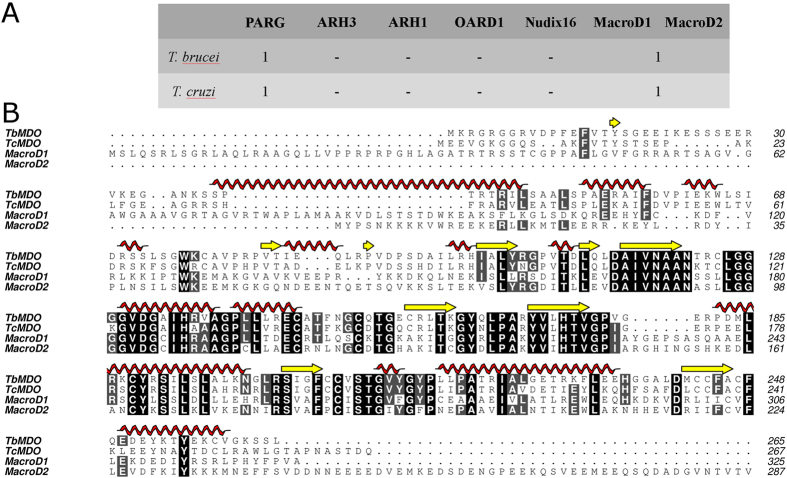
Trypanosomal ADP-ribose hydrolases. (**A**) Homologs of ADP-ribose hydrolyzing enzymes in *T. brucei* and *T. cruzi* were searched from NCBI non-redundant database using BLAST. (**B**) Sequence alignment of TbMDO, TcMDO, human MacroD1 and human MacroD2. Alignment was done using Clustal Omega^32^ and edited with Aline^33^.

**Figure 2:**
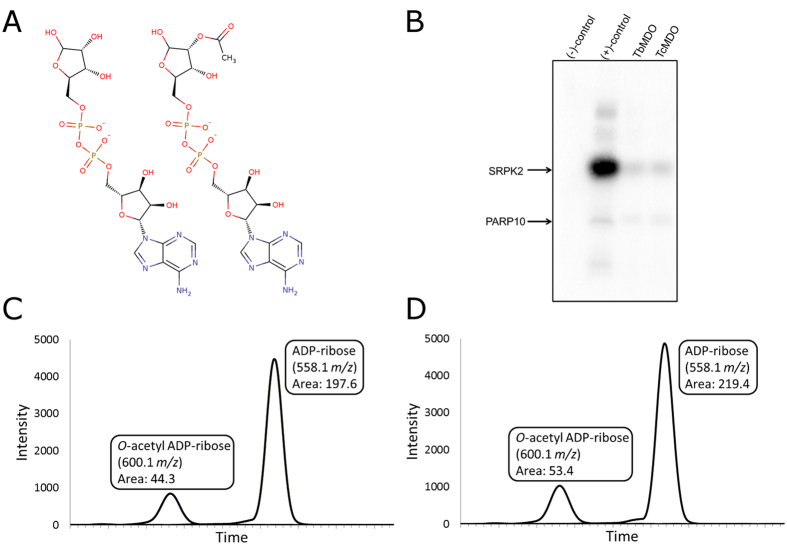
Proximal ADP-ribose and *O*-acetyl-ADP-ribose hydrolysis activity of TbMDO and TcMDO. (**A**) Structures of ADP-ribose (left) and *O*-acetyl-ADP-ribose (right). (**B**) ADP-ribose hydrolysis activity of TbMDO and TcMDO assayed by immunoblot. Negative control (−) is the reaction without biotinylated NAD^+^ and positive control (+) without incubation with the macrodomains. TbMDO and TcMDO concentration in the assay was 1 μM. (**C**,**D**) Selected ion chromatograms of the hydrolysis products of *O*-acetyl-ADP-ribose incubated with TbMDO (**C**) and TcMDO (**D**). Integrated intensities of the signals are shown in boxes.

ADP-ribose hydrolyzing enzymes of *T. cruzi* and *T. brucei* were searched from NCBI non-redundant database using known ADP-ribose hydrolases from human (PARG, ARH3, ARH1, OARD1, MacroD1, MacroD2, and Nudix hydrolase 16) as a query (Fig. 1A). Only one putative ADP-ribose hydrolase was identified from the parasites, which had homology to MacroD1 and MacroD2 (Fig. 1B). We named these proteins as *Trypanosoma brucei* MacroD-like protein (TbMDO) and *Trypanosoma cruzi* MacroD-like protein (TcMDO). It should be noted that *T. brucei* and *T. cruzi* contain putative Nudix hydrolases but they have very low identities to human Nudix hydrolase 16 and are more similar to other human Nudix proteins. Human MacroD1 and MacroD2 function as ADP-ribose hydrolases cleaving ADP-ribose from target proteins, as well as *O*-acetyl-ADP-ribose deacetylases hydrolyzing *O*-acetyl-ADP-ribose produced by sirtuins during lysine deacetylation (Fig. 2A). We assayed the ADP-ribose hydrolysis activity of the macrodomains by immunoblotting using biotinylated NAD^+^ as a substrate in PARP10 catalyzed mono-ADP-ribosylation reaction. Both proteins were able to hydrolyze the PARP10 catalyzed mono-ADP-ribosylation of an enzyme substrate, SRPK2 (Fig. 2B). The deacetylase activity was studied by incubating *O*-acetyl-ADP-ribose with the macrodomains and analyzing the hydrolysis products by mass-spectrometry (Fig. 2C,D). Incubation of *O*-acetyl-ADP-ribose with both proteins resulted in the decrease of the mass of *O*-acetyl-ADP-ribose to the molecular weight of ADP-ribose, indicating hydrolysis of the molecule.

### Thermodynamic properties of ligand binding

**Figure 3:**
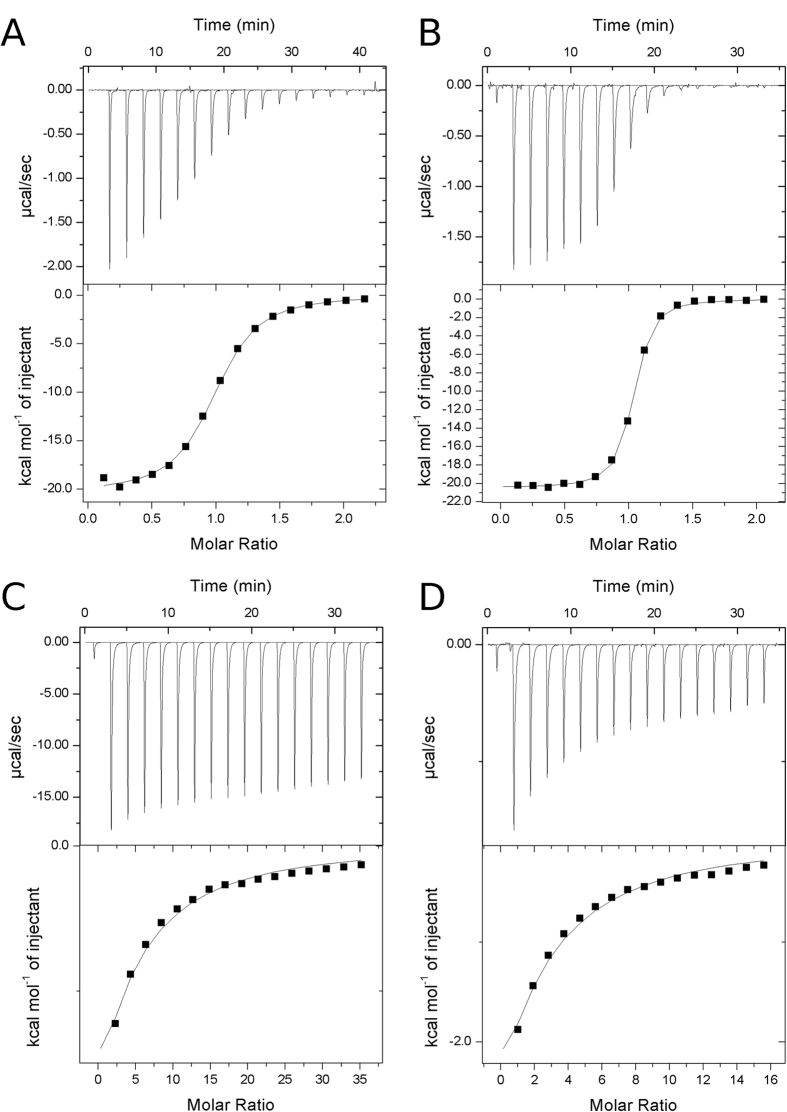
Binding of ADP and ADP-ribose to trypanosomatid macrodomains. (**A**) Titration of ADP-ribose to TbMDO, (**B**) titration of ADP-ribose to TcMDO, (**C**) titration of ADP to TbMDO and (**D**) titration of ADP to TcMDO. All titrations were performed at 25 °C. ADP-ribose titrations were repeated three times and panels (**A**,**B**) show representative titrations.

Binding energetics and affinities of ADP-ribose towards ADP-ribose hydrolases have previously been measured for human MacroD1 and MacroD2^16^. In addition, affinities of ADP-ribose and related nucleotides have been measured for *Archaeoglobus fulgidus* macrodomain Af1521^17^ and a viral macrodomain^18^. We characterized the binding of ADP and ADP-ribose to TbMDO and TcMDO with ITC (Table 1, Fig. 3). The binding of ADP-ribose to the macrodomains was driven by high enthalpy, while opposed by the entropy term. The affinities (1.3 μM and 0.2 μM for TbMDO and TcMDO, respectively) were similar to what has been observed for human MacroD1 (0.9 μM) and MacroD2 (0.15 μM)^16^.

The selectivity of the proteins towards ADP-ribose, and the importance of the ribose moiety were evaluated with ADP titrations. The distal ribose ring has a large effect on binding affinity, as the K_D_ of ADP towards the proteins was significantly increased (1.9 mM and 0.8 mM, for TbMDO and TcMDO, respectively). In the case of TbMDO, this is mainly caused by the decrease of entropy but with TcMDO the low affinity is caused solely by smaller binding enthalpy (Table 1). Altogether, the catalytic site of the trypanosomatid macrodomains is highly selective towards ADP-ribose as the exclusion of the ribose moiety results in 1500 and 4000-fold decrease in binding affinities for TbMDO and TcMDO, respectively.

### Crystal structures of trypanosomatid macrodomains

**Figure 4:**
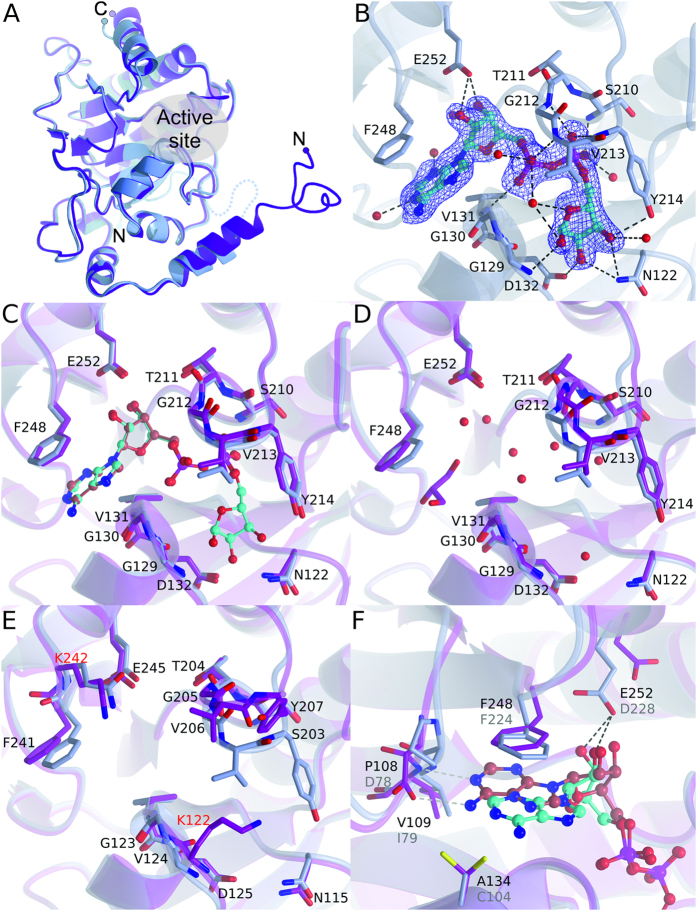
Crystal structures of trypanosomatid macrodomain proteins. (**A**) Comparison of overall structures of TbMDO (blue) and TcMDO (magenta). A disordered region in the TbMDO N-terminus is shown as a dotted line. (**B**) Close-up view of the active site of TbMDO-ADP-ribose complex. Sigma A weighted 2*F*_*o*_ - *F*_*c*_ electron density map around ADP-ribose is contoured at 1.0 σ. Hydrogen bonds are shown as black dotted lines. (**C**) Comparison ADP-ribose and ADP binding to TbMDO. TbMDO-ADP-ribose complex is shown in blue and TbMDO-ADP complex in magenta. (**D**) Comparison of the active site of TbMDO (magenta) and TbMDO-ADP-ribose complex (blue). A glycerol molecule and water molecules in TbMDO active site are shown. (**E**) Comparison of the active site of TcMDO (magenta) and TbMDO-ADP-ribose complex (blue). Residues are labelled according to TcMDO. Non-conserved residues are colored in red. (**F**) Comparison of human MacroD2-ADP-ribose complex^34^ (PDB accession code 4IQY, magenta) and TbMDO-ADP-ribose complex (blue) showing the differences in the adenine conformation. ADP-ribose is colored in red in MacroD2 and cyan in TbMDO. Non-conserved and conformationally different residues are labelled as black (TbMDO) and gray (MacroD2). Hydrogen bonds are depicted with black and gray for TbMDO and MacroD2, respectively.

Crystal structures of TbMDO were determined to 1.95 Å and 1.2 Å resolution (for crystal form 1 and 2, respectively) and crystal structure of TcMDO to 2.0 Å resolution. The two crystal forms of TbMDO are almost identical with RMSD of 0.4 Å. Structures of TbMDO and TcMDO comprise residues from 11–261 and 13–257, respectively and share a highly conserved structure (RMSD 0.84 Å). The macrodomain fold (starting from residues 99 and 92 in TbMDO and TcMDO, respectively) is well-conserved between the human MacroD proteins and consists of a central mostly parallel six-stranded β-sheet surrounded by five α-helices (Fig. 4A). RMSD of TbMDO and TcMDO to human MacroD1 are 1.1 Å and 1.4 Å and to MacroD2 1.1 Å and 1.5 Å, respectively. At the N-terminus, the macrodomains have extensions containing two α-helices, which organize in a different way in human and trypanosomatid enzymes (Fig. 4A, Supplementary Fig. 1). The N-terminal region appears to be mobile as the thermal displacement parameters in the crystal structures are high. The N-terminus of TbMDO and TcMDO also pack differently in the crystal. While the N-terminus of the TbMDO folds back to the core and extends the β-sheet, the N-terminus of the TcMDO packs to the precisely same site but in the neighboring molecule in the crystal (Supplementary Fig. 2).

### Active site

Active site of the macrodomains is located at the C-terminal edge of the β-sheet and is lined by N-terminal ends of four α-helices. The active site residues are located at the end of the helices as well as connecting loops around the site. A structure of TbMDO complexed with ADP-ribose was solved to gain insights into substrate binding. Like in MacroD2 and in Af1521 ADP-ribose is tightly coordinated in the binding site (Fig. 4B). The pocket accommodating the distal ribose is formed by two loops and especially the ribose is highly coordinated. The hydroxyls of the ribose form hydrogen bonds to surrounding Asn122, Gly129, Asp132, Tyr214, and to two water molecules. The two phosphates of the substrate are hydrogen bonded to the backbone amides of Val131, Ser210, Gly212, Val213, and Tyr214 and to three water molecules. The proximal ribose is hydrogen bonded to Glu252 and a water molecule, while the adenine moiety stacks with Phe248 and forms hydrogen bonds with two water molecules. Because of the drastically lower affinity of ADP towards the macrodomains we also determined the structure of TbMDO-ADP complex. Superposition with the ADP-ribose complex revealed that the drop in the binding affinity is a direct result in the reduction of the interactions between the ribose and the protein as the conformations of the nucleotides and coordinating residues in the active site are identical (Fig. 4C).

When comparing the ADP-ribose bound structure to apo-TbMDO, it is evident that the binding site for ADP-ribose in TbMDO is preformed (Fig. 4D). Upon binding of ADP-ribose, eight water molecules (and a glycerol molecule) are displaced but no large conformational changes in coordinating residues are observed. No complex of ADP-ribose with TcMDO could be acquired but comparing the TbMDO-ADP-ribose complex with TcMDO revealed that a loop aligning the distal ribose binding pocket adopts an open conformation, indicating that the ADP-ribose binding pocket at this region is not preformed in TcMDO (Fig. 4E). The observed open conformation is unlikely a crystal packing artefact, as there are no major constrictions caused by packing at this region of the crystal lattice. In addition, an identical conformation for the loop is observed in the apo-structure of human MacroD1 (Supplementary Fig. 3)^8^.

When compared to MacroD2 structure, the ADP-ribose site is well-conserved. Distal ribose and phosphates make extensive interactions in both structures and align well in the binding site. The major difference between TbMDO-ADP-ribose complex and MacroD2 is the conformation of the adenine moiety (Fig. 4F). Because of the proline-aspartate difference in the loop lining the adenine pocket, the moiety is unable to directly interact with the protein and is shifted 3.5 Å out from the pocket compared to MacroD2.

## Discussion

Here, we presented the identification and characterization of ADP-ribose and *O*-acetyl-ADP-ribose hydrolyzing macrodomains from *T. brucei* and *T. cruzi*. Through sequence searches we found only one conserved enzyme in the parasites potentially hydrolyzing protein-linked mono-ADP-ribosylation. These proteins had homology to human MacroD1 and MacroD2 and were named *T. brucei* MacroD-like protein (TbMDO) and *T. cruzi* MacroD-like protein (TcMDO). Importantly, the parasites seem to lack other enzymes responsible for the removal of ADP-ribose implicating highly simplified ADP-ribose metabolism in these trypanosomatids. Our qualitative analysis of the enzyme activity revealed that the macrodomains are able to hydrolyze human PARP10 catalyzed mono-ADP-ribosylation (Fig. 2B). The natural substrates for the macrodomains likely include proteins modified by ADP-ribosylating sirtuins, which have been characterized from both trypanosomatids^13^. Also proteins modified by poly-ADP-ribosylating PARPs after PARG catalyzed hydrolysis producing mono-ADP-ribosylated proteins, are potential substrates for the macrodomains. *O*-acetyl-ADP-ribose is a product of sirtuin-mediated lysine deacetylation^2^ and trypanosomatid macrodomains, similar to human MacroD1 and 2, *in vitro* hydrolyze *O*-acetyl-ADP-ribose to ADP-ribose (Fig. 2C,D). *O*-acetyl-ADP-ribose has been proposed to function as a signaling molecule in e.g. gene silencing and ion channel gating^19^, although detailed mechanisms have not been elucidated. Regardless, *O*-acetyl-ADP-ribose hydrolysis activity of the macrodomains suggests them as players in both ADP-ribose and *O*-acetyl-ADP-ribose mediated signaling in the parasites.

To scout the specificity of the ADP-ribose for the macrodomains, we measured the binding of ADP and ADP-ribose to TbMDO and TcMDO with ITC (Table 1, Fig. 3). The binding of ADP-ribose in both proteins was driven by enthalpy, also evidenced in extensive hydrogen bonding in the crystal structure of TbMDO-ADP-ribose complex (Fig. 4B). The catalytic pocket is clearly not designed to bind ADP, as the binding affinities drop significantly when the distal ribose moiety is missing. However, the thermodynamic mechanism by which the affinity decreases differs between the macrodomains. In TbMDO, the affinity decrease is mainly due to the decreased entropy, while in TcMDO the affinity drops almost exclusively because of enthalpy decrease. The larger enthalpy drop in TcMDO could be due to the open conformation of the active site (Fig. 4E). In the open conformation, the loop near the distal ribose is unable to interact with the phosphates of ADP and might explain the enthalpy change. In TbMDO, the preformed active site allows the same interactions with the phosphates of ADP as with ADP-ribose (Fig. 4C).

Structural comparison of the trypanosomatid macrodomains to human MacroD1 and 2 revealed that the core macrodomain fold is conserved. Structurally major differences can be found in the N-termini of the proteins, which are not conserved between the parasite and human proteins. The active site is well-conserved, except for the adenine-binding region, where the conformation of the adenine differs between TbMDO and MacroD2 due to a proline-aspartate change. Also TcMDO contains proline in this position indicating similar binding mode of the adenine moiety as observed in TbMDO. In humans, ADP-ribosylating enzymes in the PARP and sirtuin families are emerging targets for therapeutical intervention in several different conditions, such as cancer^20^,^21^,^22^. Also ADP-hydrolyzing enzymes have been implicated in several disease states and efforts are made to establish these proteins as therapeutic targets^23^,^24^. The involvement of PARP and PARG, the two other players in the poly-ADP-ribose synthesis and degradation, has been demonstrated in the infection process and proliferation of *T. cruzi*^12^,^14^. Most notably, iRNA and chemical inhibition of PARG leads to near complete cessation of *T. cruzi* infection^14^. Currently there are no inhibitors against macrodomain proteins available complicating the analysis of their cellular functions but the seemingly simple ADP-ribose metabolism of trypanosomatids might be amenable to targeting by antiparasitic drugs.

## Methods

### Cloning and expression

Codon optimized cDNAs of *Trypanosoma brucei gambiense* macrodomain (Uniprot id C9ZP98) and *Trypanosoma cruzi* (strain CL Brener) macrodomain (Uniprot id Q4DQ03) were purchased from GenScript (NJ, USA). DNA encoding for the full-length proteins were cloned to pNH-Trxt vector *via* PCR extension cloning. Proteins were expressed in *E. coli* BL21 (DE3) cells grown in Terrific broth auto-induction media (Formedium) supplemented with 8 g/l glycerol and 50 μg/ml of kanamycin. The cells were grown at 37 °C until OD_600_ reached 1.0 and the temperature was lowered to 20 °C for protein expression. After 20 hours the cells were collected, suspended in lysis buffer (50 mM HEPES pH 7.4, 0.5 M NaCl, 10% glycerol, 0.5 mM TCEP) and stored at −20 °C.

### Protein purification

Cell suspension was supplemented with 0.5 mg/ml lysozyme, 20 μg/ml DNaseI and 0.1 mM Pefabloc SC (Sigma-Aldrich). Cells were lysed by sonication and the cell debris was cleared by centrifugation (31000 × *g*, 45 min at 4 °C). Supernatant was filtered through a 0.45 μm filter and loaded on a 1 ml HisTrap Chelating HP column (GE Healthcare) charged with Ni^2+^. The column was subsequently washed with lysis buffer and wash buffer (50 mM HEPES pH 7.4, 0.5 M NaCl, 10% glycerol, 25 mM imidazole, 0.5 mM TCEP). Proteins were eluted with 50 mM HEPES pH 7.4, 500 mM NaCl, 10% glycerol, 500 mM imidazole, 0.5 mM TCEP. The fusion-tags were cleaved with TEV-protease at 4 °C overnight. The solutions were loaded again on a Ni^2+^ charged 1 ml HisTrap Chelating HP column and the proteins were collected from the flowthrough. The proteins were further purified with HiLoad 16/600 Superdex 75 pre-equilibrated with gel filtration buffers (TbMDO: 20 mM HEPES pH 7.0, 300 mM NaCl, 0.5 mM TCEP; TcMDO: 20 mM HEPES pH 8.0, 300 mM NaCl, 0.5 mM TCEP). The purified proteins were flash-frozen in liquid N_2_ and stored at −70 °C. PARP10 and SRPK2 were expressed and purified as described earlier^25^.

### Immunoblotting

Proximal ADP-ribose hydrolysis activity of the macrodomains was tested with immunoblotting. PARP10 (0.5 μM) and SRPK2 (2 μM) were incubated in 50 mM Tris pH 7.0 with or without 1 μM biotinylated NAD^+^ (Trevigen, USA) at 22 °C for three hours. Then 1 μM of TbMDO or TcMDO was added to the reaction and the incubation was continued for another three hours. The reactions were stopped by adding 2× Laemmli buffer (Bio-Rad, USA) and heating the mixture at 95 °C for 5 min. The samples were separated by SDS-PAGE and transferred onto a nitrocellulose membrane (Whatman, UK). The membrane was blocked using 1% Casein in 1xTBS (Bio-Rad, USA) and visualized with 1:15000 streptavidin conjugated horseradish peroxidase (PerkinElmer, USA) and chemiluminescent substrate (WesternBright^TM^ ECL, Advansta Corporation, USA).

### Mass spectrometry

*O*-acetyl-ADP-ribose hydrolysis activity of the macrodomains was analyzed by incubating 0.5 mM of 2′/3′-*O*-acetyl-ADP-ribose (Santa Cruz Biotechnology) with 50 μM TbMDO or TcMDO at 22 °C for 4 hours. The proteins were precipitated with 80% (v/v) acetone and the precipitation was removed by centrifugation. The reactions were loaded onto an Acquity UPLC BEH C18 2.1 × 100 mm, 1.7 μm column (Waters) and signals were analyzed by mass spectrometry (ESI-TOF). A control measurement was done with 2′/3′-*O*-acetyl-ADP-ribose without incubation with the macrodomains. *O*-acetyl-ADP-ribose control did not show significant signal over background for ADP-ribose (558.1 *m/z*).

### Isothermal titration calorimetry

The binding of TbMDO and TcMDO to ADP and ADP-ribose was analyzed by isothermal titration calorimetry using MicroCal iTC200 (Malvern) at 25 °C. The titrations were performed in 50 mM HEPES pH 7.5, 250 mM NaCl, 0.5 mM TCEP with 40–150 μM of protein and 0.4–25 mM of nucleotide. Dilution heats were measured and subtracted from the binding isotherms in both ADP titrations and TbMDO-ADP-ribose titrations. Residual dilution heats were directly subtracted from binding isotherms in the TcMDO-ADP-ribose titrations. Due to very low binding affinity, full binding isotherm was not obtained for the ADP titrations and these data were analyzed by constraining binding stoichiometry (n) to 1. ADP-ribose titrations were repeated three times and ADP titrations were performed once. Data were analyzed with Origin 7 (OriginLab).

### Protein crystallization

All crystallizations were done using a sitting-drop vapor-diffusion method at 20 °C. TbMDO (21.5 mg/ml) was crystallized in two crystal forms. Crystal form 1 (P6_1_22) was obtained with 0.1 M Bis-Tris propane pH 7.5, 0.2 M sodium sulfate, 20% w/v PEG 3350. Crystal form 2 (C2) crystallized in 0.1 M Bis-Tris propane pH 8.5, 0.2 M sodium fluoride, 20% w/v PEG 3350, 2.8 mM AMP.

TbMDO was co-crystallized with ADP in 0.1 M MIB (malonic acid, imidazole, boric acid) pH 8.0, 25% w/v PEG 1500, 2.8 mM ADP. TbMDO – ADP-ribose complex was crystallized in 0.1 M Bis-Tris propane pH 8.5, 0.2 M sodium fluoride, 20% w/v PEG 3350, 1.7 mM ADP-ribose. TbMDO was also co-crystallized with 2′/3′-*O*-acetyl-ADP-ribose but only ADP-ribose was observed in the electron density. We also soaked 2′/3′-*O*-acetyl-ADP-ribose to preformed crystals but again only ADP-ribose was visible in the electron density maps.

TcMDO (16.6 mg/ml) was crystallized in 0.1 M Bis-Tris propane pH 8.0, 0.96 M trisodium citrate. TcMDO co-crystallization and soaking was attempted with AMP, ADP, ADP-ribose and 2′/3′-*O*-acetyl-ADP-ribose. However, co-crystallization did not result in crystal formation and soaking destroyed any diffraction from the crystals.

Prior to data collection, crystals were quickly soaked in crystallization solution supplemented with 20% glycerol and flash frozen in liquid nitrogen.

### Crystallographic data collection, processing and refinement

Diffraction data were collected on beam lines I03 and I04 at Diamond Light source, Didcot, UK. Data were processed and scaled with the XDS package^26^. Phases for TbMDO crystal form 1 were obtained by molecular replacement with Phaser^27^ using a truncated poly-Ala model of human MacroD1 (PDB accession code 2X47) as a search model. Phases for the crystal form 2 and TbMDO-ADP and TbMDO-ADP-ribose complexes were obtained by molecular replacement with Phaser using a monomer from the crystal form 1 as a search model. TcMDO structure was determined by molecular replacement with Phaser using the apo-TbMDO crystal form 1 as a search model. ARP/wARP^28^ was used for automatic model building of TbMDO crystal form 1 and TcMDO. The structures were refined with REFMAC5^29^ and phenix.refine^30^. Coot^31^ was used for visualization and model building. Data collection and refinement statistics are shown in Table 2.

## Additional Information

**How to cite this article**: Haikarainen, T. and Lehtiö, L. Proximal ADP-ribose Hydrolysis in Trypanosomatids is Catalyzed by a Macrodomain. *Sci. Rep.* **6**, 24213; doi: 10.1038/srep24213 (2016).

## Supplementary Material

Supplementary Information

## Figures and Tables

**Table 1:** Thermodynamic parameters of ADP and ADP-ribose binding.

	n	ΔH	−TΔS	ΔG	K
**ADP-ribose**	±SEM	±SEM (kcal/mol)	(kcal/mol)	(kcal/mol)	±SEM (×10^6 ^M^−1^)
TbMDO	0.97 ± 0.01	−20.0 ± 0.5	12.0	−8.0	0.79 ± 0.04
TcMDO	1.0 ± 0.02	−20.1 ± 0.3	10.9	−9.1	4.87 ± 0.09
**ADP**		(kcal/mol)	(kcal/mol)	(kcal/mol)	(×10^3 ^M^−1^)
TbMDO	1	−18.9	15.2	−3.7	0.54
TcMDO	1	−14.8	10.6	−4.2	1.22

Three titrations were performed with ADP-ribose and n, ΔH and K are reported with SEM.

**Table 2:** Data processing and refinement statistics.

PDB code	apo-TbMDO crystal form 1	apo-TbMDO crystal form 2	TbMDO-ADP-ribose	TbMDO-ADP	TcMDO
	5FSU	5FSV	5FSY	5FSX	5FSZ
**Data**					
Beam line	Diamond I03	Diamond I03	Diamond I03	Diamond I03	Diamond I04
Wavelength (Å)	0.97625	0.97625	0.97625	0.97625	0.97949
Space group	P6_1_22	C2	C2	I2	P4_2_2_1_2
Cell dimensions					
a, b, c (Å)	77.14, 77.14, 427.45	78.39, 54.54, 58.82	78.05, 54.62, 58.76	65.96, 115.59, 70.44	74.82, 74.82, 75.44
Resolution (Å)	30–1.95 (2.00–1.95)	30–1.2 (1.23–1.20)	30–1.7 (1.74–1.70)	30–2.0 (2.05–2.00)	30–2.0 (2.05–2.00)
R_merge_	0.093 (3.161)	0.045 (0.853)	0.062 (1.342)	0.065 (1.135)	0.128 (1.681)
I/σI	16.9 (1.8)	11.9 (1.5)	10.8 (1.1)	11.5 (1.6)	10.7 (1.5)
Completeness (%)	99.7 (99.7)	99.5 (99.7)	96.3 (98.9)	96.9 (96.6)	99.8 (100)
Redundancy	15.1 (15.1)	3.2 (3.0)	3.1 (3.1)	3.4 (3.6)	10.4 (11.1)
**Refinement**					
Reflections	53658	72804	24644	34678	14251
R_work_/R_free_	0.189/0.219	0.144/0.160	0.201/0.244	0.214/0.253	0.188/0.258
B-factors					
Protein	54.0	18.6	30.9	61.5	43.3
Ligand	–	–	27.8	53.1	–
R.m.s.d.					
Bond lengths (Å)	0.011	0.010	0.013	0.008	0.010
Bond angles (°)	1.4	1.5	1.7	1.2	1.4

Values for the highest resolution shell are shown in parentheses.
