# Supplementary material for: Proximal ADP-ribose Hydrolysis in Trypanosomatids is Catalyzed by a Macrodomain
Source: Sci Rep. 2016 Apr 11;6:24213. doi: 10.1038/srep24213 (PMC4827093; doi:10.1038/srep24213)
Supplement: Supplementary Information [file srep24213-s1.pdf]

Supplementary Information

Proximal ADP-ribose Hydrolysis in Trypanosomatids is Catalyzed by a Macrodomain

Teemu Haikarainen[1], & Lari Lehtiö[1,*]

[1]Biocenter Oulu and Faculty of Biochemistry and Molecular Medicine, University of Oulu, FI-90014 Oulu, Finland
*Corresponding author: Lari Lehtiö (lari.lehtio@oulu.fi)

Content:

Supplementary Figure 1
Supplementary Figure 2
Supplementary Figure 3

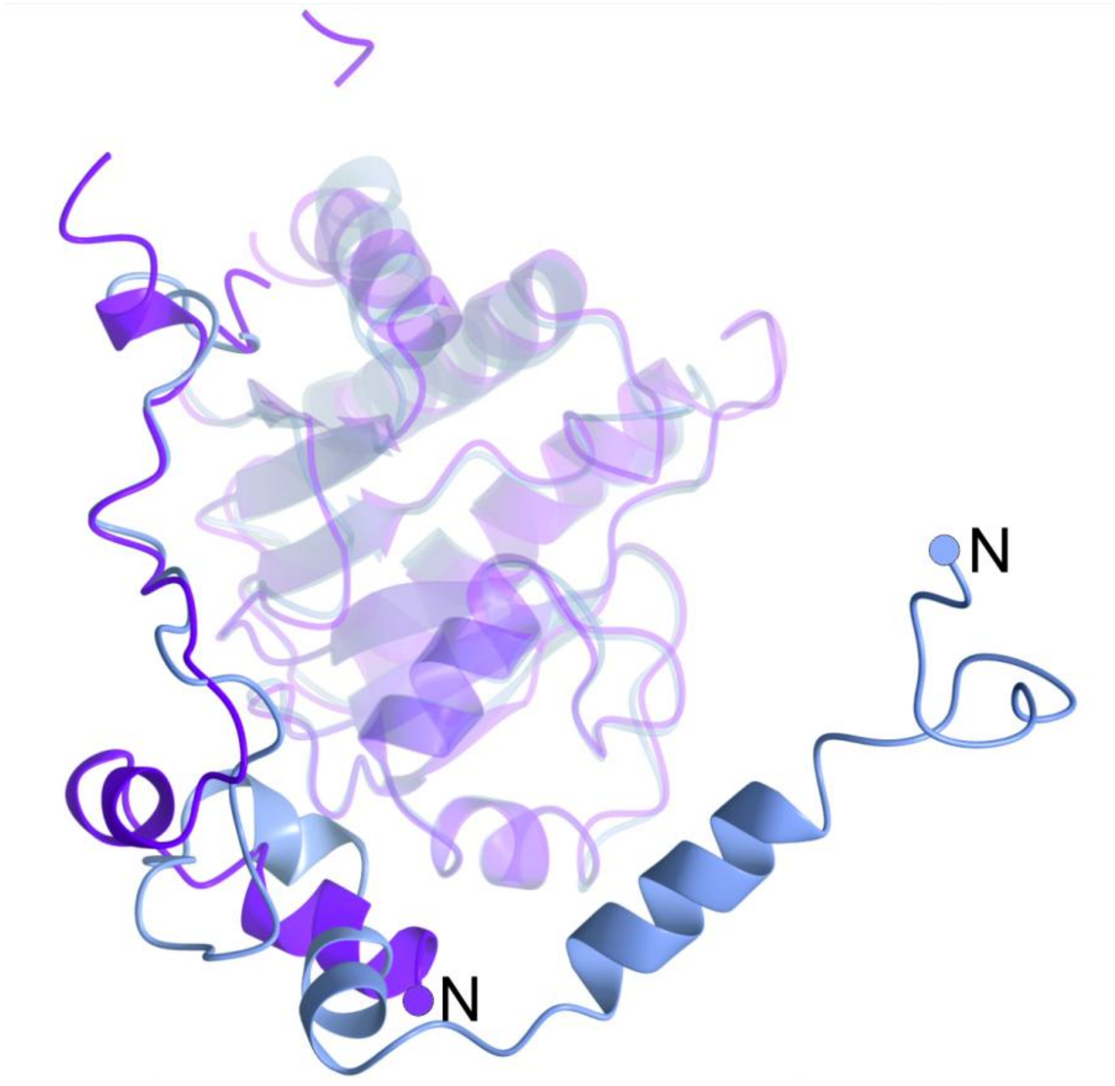

Supplementary Figure 1. Organization of the N-terminus of TcMDO (blue) and human MacroD2 (magenta). The N-termini of the proteins are shown in front, while the conserved macrodomain fold is shown in the background.

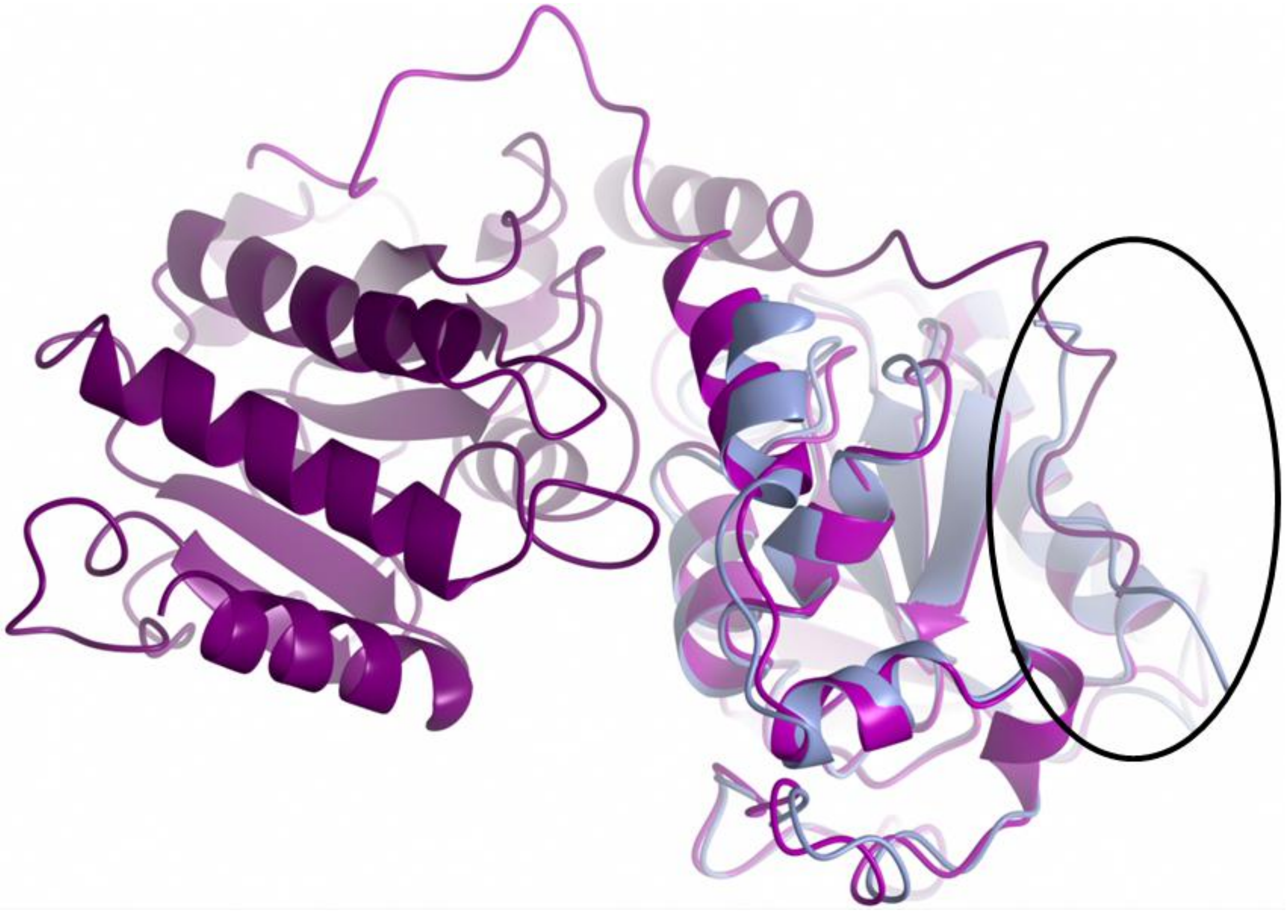

Supplementary Figure 2. Packing of the N-termini of the macrodomains. Superposition of TbMDO (blue) with TcMDO (magenta). N-terminus of the symmetry related TcMDO molecule (dark magenta) packs in identical location as the N-terminus of TbMDO (circled).

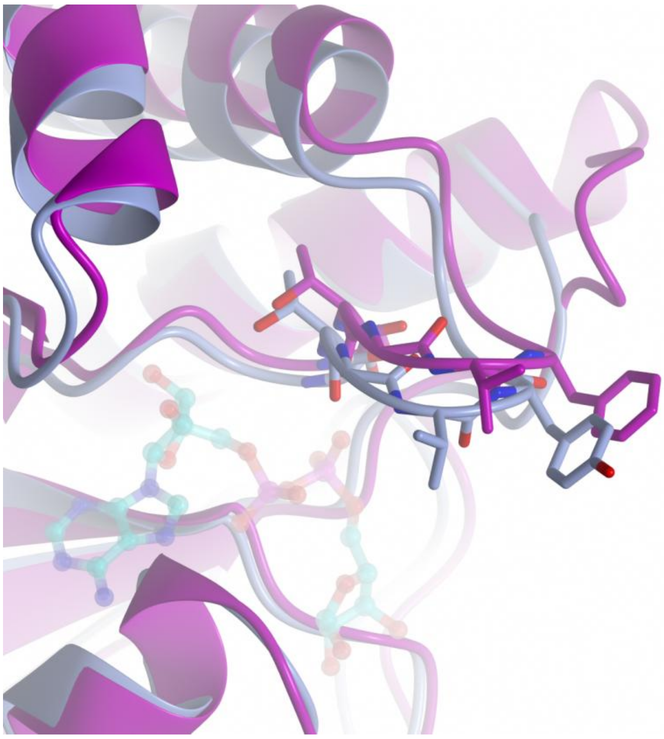

Supplementary Figure 3. Conformation of the loop next to distal ribose binding pocket in TcMDO. Superposition of the TcMDO (blue) and MacroD1 (magenta). ADP-ribose from the TbMDO-ADP-ribose complex is shown in the background to illustrate the binding site.
